# Efficacy of Nutritional Interventions as Stand-Alone or Synergistic Treatments with Exercise for the Management of Sarcopenia

**DOI:** 10.3390/nu11091991

**Published:** 2019-08-23

**Authors:** Sarah Damanti, Domenico Azzolino, Carlotta Roncaglione, Beatrice Arosio, Paolo Rossi, Matteo Cesari

**Affiliations:** 1 Geriatric Unit, Fondazione IRCCS Ca’ Granda Ospedale Maggiore Policlinico, 20122 Milan, Italy; 2 Phd Course in Nutritional Sciences, University of Milan, 20122 Milan, Italy; 3 Department of Clinical Sciences and Community Health, University of Milan, 20122 Milan, Italy

**Keywords:** sarcopenia, nutrition, physical exercise, synergism, older people

## Abstract

Sarcopenia is an age-related and accelerated process characterized by a progressive loss of muscle mass and strength/function. It is a multifactorial process associated with several adverse outcomes including falls, frailty, functional decline, hospitalization, and mortality. Hence, sarcopenia represents a major public health problem and has become the focus of intense research. Unfortunately, no pharmacological treatments are yet available to prevent or treat this age-related condition. At present, the only strategies for the management of sarcopenia are mainly based on nutritional and physical exercise interventions. The purpose of this review is, thus, to provide an overview on the role of proteins and other key nutrients, alone or in combination with physical exercise, on muscle parameters.

## 1. Sarcopenia

Muscle mass and strength progressively decline after the age of 40. Then, these age-related changes substantially accelerate after the age of 60, especially in the presence of sedentary behavior and comorbidities [1]. This clinical manifestation of aging is called sarcopenia (from the Ancient Greek σάρξ (sárx, “flesh”) + πενῐ́ᾱ (peníā, “poverty”), and has recently been the object of increasing attention from researchers, clinicians, and public health authorities. Its growing relevance is paradigmatically exemplified by the recent inclusion of a specific ICD-10 code for it [2].

Contrary to lean mass decline, there is an increase in fat mass, with aging [3]. Adipose tissue can infiltrate muscles both at macroscopic (between muscle groups) and microscopic level (between and inside myocytes) with a further reduction in muscle mass and quality [4].

Sarcopenia represents an important sanitary problem since it affects 20% of people over 70 and 50% of people over 80 [5]. Moreover, considering the important function of muscle tissue beyond locomotion (e.g., influence on glucose and protein metabolism and on bone density) [6], it is associated with many adverse clinical outcomes (falls, fractures, functional and cognitive decline, cardiac and respiratory disease, reduced quality of life and independence, hospitalization, and mortality) [7,8,9,10,11,12,13,14,15,16,17,18,19]. Therefore, an early detection and treatment of this condition is pivotal.

In recent years, many studies have consistently demonstrated that muscle strength declines more rapidly (1.5%–5% year after the age of 50) than muscle mass (1%–2% year after the age of 50) [1,20,21,22,23,24,25,26,27], allowing an earlier identification of muscle impairment. Moreover, muscle strength correlates better than muscle mass with adverse health outcomes [28]. Therefore, in 2009, the European Working Group on Sarcopenia in Older People (EWGSOP) met to elaborate a definition of sarcopenia, which included both the presence of low muscle mass and function [29]. Sarcopenia was defined as a geriatric syndrome characterized by a progressive loss of muscle mass and strength. However, recently this definition has been updated [30] in light of the recognition of the role as disease of sarcopenia and of the scientific advantages, which stress the role of muscle strength as a principal determinant of the condition. Sarcopenia is now defined as a muscle disease, which can be considered probable if reduced muscle strength is detected. The main tools used to assess muscle strength are the hand grip dynamometer [31] or the chair stand test [32,33]. The diagnosis is confirmed in the presence of reduced muscle mass. The main instrument to assess muscle mass used for research purpose is the dual-energy X-ray absorptiometry (DXA). Bioelectrical impedance analysis [34] is another possibility, though less precise and more sensitive to body water content changes. Finally, in selected settings (i.e., oncologic patients), abdominal CT scans [35] performed for other purposes can be employed to estimate body muscle mass. Instead, anthropometric measures (i.e., calf circumference), though easy to assess, are not considered a reliable measure of muscle mass [36].

The severity of the condition can be then graded by measuring muscle performance. The most common used tools are the Short Physical Performance Battery32, the timed ‘up and go’ test [37], and the 400 m walking test [38].

Several mechanisms concur to the development of sarcopenia, including malnutrition, hormonal changes (i.e., reduction of growth hormone, estrogens, and testosterone), increased production of pro-inflammatory cytokines (also by the adipose tissue infiltrating the muscle [9]), higher muscle protein breakdown, myocytes loss, reduced satellite cell replenishment, loss of α-motor neurons, muscular mitochondrial dysfunction, altered myocyte autophagy, accelerated apoptosis of myonuclei, and impaired satellite cell function. Recently, the role of the fibromodulin has been highlighted, which is an extracellular matrix protein and predominantly controls a wide range of myogenesis-related genes (i.e., myogenin, myosin light chain 2, and transcriptional activity of myostatin) [39,40]. Fibromodulin is an essential part of the myogenic program and its role in the regulation of myoblasts may help in the development of new therapeutic agents (i.e., novel myostatin inhibitors) for the treatment of different muscle atrophies like as sarcopenia [39,40]. Furthermore, insulin resistance reduces the ability to use the available proteins [41]. Insulin resistance in the skeletal muscle results in whole-body metabolic disturbances associated with type 2 diabetes, which are further exacerbated by sarcopenia [42,43]. All these alterations are differently responsible for an imbalance between the anabolic and catabolic process at the muscular level [44]. Nevertheless, this list of pathophysiological pathways has to be considered as non-exhaustive, especially because novel mechanisms are under study and continuously propose novel/complementary possibilities. Pathophysiological mechanisms represent a possible target for therapeutic interventions.

## 2. Nutritional Interventions

Malnutrition is a state resulting from the lack of intake or uptake of nutritional elements, which alters body composition and body cell mass. Its etiologies can be heterogeneous: Starvation [45], cachexia [46], or simply aging [47]. Malnutrition has serious consequences, thus impairing both physical and mental functions and predisposing to adverse clinical outcomes from diseases [48]. Indeed, reduced intake of specific nutrients compromises the anabolic signal to muscles whereas their altered uptake configures a situation of anabolic resistance. Both conditions contribute to the development of sarcopenia [49].

Recently, the European Society of Clinical Nutrition and Metabolism (ESPEN) has validated the new diagnostic criteria for malnutrition [50]. The diagnosis can be performed if body mass index (BMI) is <18.5 kg/m^2^ or if an unintentional weight loss is associated with either a reduced BMI (<20 kg/m^2^ in younger or <22 kg/m^2^ in older patients) or a low-fat free mass index. Moreover, malnutrition should be screened in all individual who are potentially at risk.

### 2.1. Proteins and Essential Amino Acids

Dietary proteins stimulate skeletal muscle protein synthesis and inhibit muscle protein breakdown [51,52,53]. Some observational studies have showed an association between protein intake and muscle mass and strength [54,55,56].

The effect of protein supplementation has been particularly evident on muscle strength and function [57] rather than on mass. However, protein supplementation alone could not be sufficient in cases of severe catabolism [58].

Older people frequently fail to reach the recommended dietary allowance (RDA) of proteins and calories. First of all, there is a reduction in appetite with aging, the so-called “anorexia of aging” [59,60]. Moreover, eating habits change due to swallowing and/or economic problems. Thus, the consumption of proteins rich nutrients switches in favor of energy-dilute foods (grains, vegetables, and fruits) [61].

Recently, two consensus studies (ESPEN [62] and PROT-AGE study group [41]) have stated that the traditional RDA of proteins for adults of all ages (0.8 g/kg body weight/day [63]) was not sufficient for older people. People aged 65 and older need more proteins to activate the muscle protein synthesis, compared to younger people [41,64]. Actually, older people have to counteract an anabolic resistance underpinned by an increased splanchnic sequestration of amino acids, lower postprandial perfusion of muscles, decreased muscle uptake of dietary amino acids, reduced anabolic signaling for protein synthesis, and an impaired digestive capacity [41,65,66]. Moreover, they require more proteins to offset inflammatory and catabolic conditions associated with chronic and acute diseases [67]. Thus, both the ESPEN and the PROT-AGE group concord in suggesting the assumption of 1–1.2 g proteins/kg body weight/day. A high-protein diet does not damage the kidney in healthy old individuals [68,69], whereas people with a severe kidney disease who do not undergo dialysis should limit their protein intake at about 0.8 g/kg body weight/day [41].

The protein source and amino acid composition are also important: Plant proteins have a lower anabolic effect compared to animal proteins [70], probably because they have a lower content of leucine. Moreover, independently from the amino acid content, proteins could have different absorption kinetics, which could influence their anabolic effect. There is a debate whether slow or fast digested proteins could better influence a muscle synthetic response [71,72,73]. It seems that fast proteins are more effective in stimulating muscle protein accretion41 even if results should be confirmed in larger trials.

Spread feeding patterns, in which an equal amount of protein is ingested at each meal, seems to optimize the protein synthetic capacity [74]. Nevertheless, some studies have also showed that with pulse feeding (with a main high protein meal), anabolic benefits can be reached [75,76]. Thus, additional trials are needed to establish the optimal timing of protein administration.

Moreover, some proteins are metabolized to short-chain fatty acids (like propionate, butyrate, and acetate) which are used by muscle cells to produce energy [77,78,79,80]. Indeed, short-chain fatty acids promote muscle anabolism and display anti-inflammatory proprieties positively, influencing muscle health [81,82,83,84].

Essential amino acids (EAAs), in particular leucine, are an important anabolic stimulus, too [85]. The main dietary sources of EEAs are lean meat, dairy products, soybeans, cowpeas, and lentils. The biological pathways on which leucine act are the activation of the mammalian target of rapamycin (mTOR) [86] and the inhibition of the proteasome [87]. However, supplementation with high doses of EEAs (10–15 g) and leucine (at least 3 g) is necessary to overcome anabolic resistance in older people [88].

A recent meta-analysis has confirmed that leucine is able to increase muscle protein synthesis in older people [89] and its consumption has been found to be directly correlated with muscle mass retention in healthy older people [90]. What is more, the supplementation with EAA in older people has been effective in increasing both muscle mass and function [91].

### 2.2. β-Hydroxy β-Methylbutyrate

ß-hydroxy ß-methylbutyrate (HMB) is one of the metabolites of leucine, which exerts anabolic effects through the activation of the mTOR pathway and the stimulation of the growth hormone/IGF-1 axis [92]. HMB also has anticatabolic effects: It decreases ubiquitin-proteasome expressions [93,94] and attenuates the up-regulation of caspases [95]. Moreover, it increases mitochondrial biogenesis and fat oxidation [96], possibly contributing to the improvement of muscle performance. HMB also favors sarcolemmal integrity via its conversion into hydroxymethylglutaryl-CoA [97]. Therefore, its final effects are induction of myogenic proliferation and differentiation [98]. Only 5% of leucine is usually metabolized to HMB [99] and there is an age-related decline in its concentrations [100]. Thus, there is a rationale for supplementation with HMB in old sarcopenic individuals. HMB is frequently used by athletes to better their physical performance [101]. It has been shown to be effective in improving muscle mass and strength in older adults too. What is more, HMB contributes to the preservation of muscle mass during bed rest, a known wasting condition [102,103,104,105].

### 2.3. Ornithine α-Ketoglutarate

Ornithine α-ketoglutarate (OKG) is a compound formed by ornithine (a non-proteinogenic amino acid) and α-ketoglutarate (a keto acid derived from the deamination of glutamate). It can offset sarcopenia through various mechanisms. OKG is the precursor of many amino acids (glutamate, glutamine, arginine, and proline), and of nitric oxide (NO), which improves hemodynamics in skeletal muscles [106]. Furthermore, it acts as a secretagogue of anabolic hormones like insulin and the growth hormone [106,107]. Therefore, OKG has successfully been used in experimental conditions of hypercatabolism (e.g., malnutrition, cancer cachexia, burn injury, and surgery) to reduce muscle wasting [108,109,110,111]. However, its effects have not been investigated yet, in non-malnourished older people.

### 2.4. Vitamin D

Deficit of vitamin D has been associated with reduced muscle mass and strength in prospective studies [112,113]. Most older people have serum levels of vitamin D below the normal range. The etiology of this deficit is multifactorial: Insufficient dietary intake, inadequate sunshine exposure, altered skin synthesizing capacity, and diminished renal conversion to the active form [114]. Moreover, there is a reduction in the expression of vitamin D receptors on muscle tissue [115,116] with aging.

The effects of vitamin D are mediated by its link with nuclear (VDR) and membrane-bound vitamin D receptors. The former activate the transcription of target genes involved in calcium uptake, phosphate transport, satellite cells proliferation, and terminal differentiation [117] while the latter regulate the release of calcium into the cytosol, respectively. This is pivotal for muscle contraction and induces protein synthesis [118].

Vitamin D supplementation can modulate the expression of VDR [119] with positive effects on muscle performance and strength [120,121]. What is more, it also improves muscle fiber composition and morphology [122]. Curiously, benefits seem to be appreciable only in people with low levels of vitamin D.

Therefore, it is recommended to dose vitamin D in all sarcopenic patients and to prescribe supplements in those who are deficient. Promotion of an adequate sunshine exposure together with the consumption of foods rich in vitamin D (salmon, mackerel, herring, sun-dried mushrooms) should instead be suggested in all older people.

### 2.5. Creatine Monohydrate

Creatine (Cr) is a compound that can be assumed with food (lean red meat, tuna, and salmon) or can be synthetized endogenously in the liver and kidneys using the amino acids glycine, arginine, and methionine.

Most of the creatine is stored in the skeletal muscles where it is converted into a high-energy metabolite phosphocreatine (PCr) by the enzyme creatine kinase. PCr acts as an energy buffer: At the beginning of muscular contraction, it donates a phosphate group to ADP to form ATP in order to produce energy anaerobically. During rest, the opposite process takes place and the excess of ATP is used to regenerate PCr from Cr [123,124].

Moreover, Cr activates the transcription of genes involved in muscle protein synthesis and satellite cells activation probably mediated by the mTOR pathway [125,126,127]. In fact, creatine may enhance muscle mass and force probably by increasing the expression of IGF-1 [127,128], which seems to activate the key elements of protein synthesis of the IGF1-IRS1-PI3K-AKT-mTOR pathway [129,130,131]. Indeed, it is well known that the activation of the IGF1-IRS1-PI3K-AKT-mTOR pathway induces muscle hypertrophy [132,133]. The consequent increase of IGF-1 via Cr is also observable in the significantly increased expression of several myogenic regulatory factors (i.e., Myo-D, Myf-5 and MRF-4) [128], which are responsible for satellite cell activation, proliferation, and differentiation [134]. This positive effect of creatine on muscle is probably only observable together with exercise [135,136,137]. Just recently, Ferretti et al. [138] demonstrated that during resistance training, Cr monohydrate increases muscle size and performance, suggesting a higher activation of muscle protein synthesis via IGF1-IRS1-PI3K-AKT-mTOR pathway.

Considering that in older people intramuscular Cr levels are reduced [139], the supplementation with Cr could be very beneficial. Indeed, there is evidence that high consumption of creatine can improve muscle mass and functions in older people [140].

### 2.6. Antioxidants and polyunsaturated fatty acids

Aging is characterized by oxidative stress-induced damages in various organs and systems [141].

In the pathogenesis of sarcopenia, there is an oxidative damage to muscle mitochondria and membranes, which compromises ATP production and increases sarcolemma permeability. Both alterations cause energy deprivation and activate stress pathways, which lead to muscle cell apoptosis [142,143,144,145].

The base of this damage is an imbalance between reactive oxygen species production and antioxidant defenses. Supplementation of exogenous antioxidants has been proposed in older people, since it can help the action of endogenous antioxidant enzymatic systems (i.e., superoxide dismutase, glutathione peroxidase)

It is true that high-plasma carotenoids have been associated with a lower risk of developing walking disability and decline in muscle strength in community-dwelling older people [146,147]. Anyway, excessive vitamin C and E supplementation can compromise muscular adaptations to strength training in older people [148]. This is because a limited ROS production is pivotal to promote adaptation to exercise [149]. In fact, it favors force production and mitochondrial biogenesis [150,151]. Excessive antioxidant supplementation in people who are not deficient could therefore compromise the mechanism of adaption to exercise. Thus, the supplementation can have a final negative effect on muscle mass and performance.

Moreover, many antioxidants (like selenium, vitamin A, vitamin C, vitamin E, and β-carotene) can also behave as potent pro-oxidants under some circumstances [152]. This is one more reason why supplementation with antioxidant in people who are not deficient can blunt the beneficial effects of physical exercise [153]. Meta-analyses and systematic reviews have demonstrated an increased mortality in both healthy people and those with various diseases who have been supplemented with antioxidants [154,155,156]. Considering the possible harms of antioxidant supplementation, its use to prevent or treat sarcopenia should be avoided unless an overt deficit is documented [157]. Instead, promoting the regular consumption of foods naturally rich in antioxidant could be beneficial in older people [158].

Supplementation with polyunsaturated fatty acids (PUFAs), and in particular with omega-3 fatty acids, improves muscle protein anabolism. PUFAs seem to directly act on mTOR signaling [159] and reduce inflammation [160,161]. The main dietary source of PUFAs is fatty fish: Salmon, mackerel, herring, lake trout, sardines, albacore tuna, and their oils. A higher dietary consumption of PUFAs has been associated with a greater fat-free mass [162]. A positive correlation between fatty fish (which is rich in PUFA) and grip strength was found in community-dwelling older people [163] too.

However, many studies on supplementation with PUFAs (with different dosages and for different periods) have produced different but mainly inconsistent results so far [164,165]. Moreover, the risk for potential adverse events associated with long-term supplementation has not been clearly elicited. Therefore, there is insufficient evidence to promote the systematic consumption of PUFAs in sarcopenic individuals.

### 2.7. Ursolic Acid

Ursolic acid is a compound with anti-inflammatory properties, which is abundant in apple peels, plum, cranberry, blueberry, rosemary, hawthorn, thyme, basil, oregano, and peppermint [166].

In murine models, ursolic acid has displayed anabolic proprieties mediated by the repression of atrophy-associated genes (atrogin-1 and MuRF1), and the induction of trophic genes (PKB/Akt and S6 kinase). Furthermore, it was able to stimulate the insulin/IGF-1 axis producing muscular hypertrophy [167]. However, supplementation in sarcopenic individuals has not been performed yet.

### 2.8. Nitrate-Rich Foods

Nitrate (NO3–)-rich foods (e.g., celery, cress, chervil, lettuce, red beetroot, spinach, and rocket) have a potential role in the treatment of sarcopenia. Food-derived NO3− is reduced to NO2− by commensal bacteria of the oral cavity [168]. Through several mechanisms, NO2– is then converted to nitric oxide (NO), which is the active mediator of the anti-sarcopenic effects of these compounds. The increase in gastric levels of NO attenuates the aging anorexia by reducing the earlier satiety feeling [59]. Furthermore, by improving the endothelial function, NO improves nutrient supply to muscles [169].

Finally, NO optimizes mitochondrial bioenergetics, by reducing the metabolic cost of exercise [170,171]. Indeed, NO was effective in improving muscular performance in young individuals [172]. On the contrary, short-term supplementation with nitrate-rich foods did not improve muscular performance and strength [173] in older people. Thus, there has been insufficient evidence so far to recommend the supplementation with nitrate-rich foods in sarcopenic individuals.

### 2.9. Prebiotics, Probiotics, and Symbiotics

Recently, gut microbiota has been proposed as a contributor in the pathogenesis of sarcopenia, so interventions that promote its health can be beneficial. Older people tend to develop an intestinal dysbiosis, which is associated with an increased gut permeability. Alterations of the gut barrier facilitate the passage of endotoxins and other microbial products with inflammatory effects into the blood stream. This contributes to the development of a deleterious state of systemic inflammation [174] contributing to muscle wasting [78,175].

Moreover, the reduction of intestinal mobility, typical of older persons, also alters the species of bacteria colonizing the gut (*Bacteroidetes* and *Firmicutes*) in favor of species with greater proteolytic potential (*Proteobacteria*) [176], consequently influencing the proper utilization of dietary proteins [175]. An enhanced proteolytic capacity and anabolic resistance of the gut microbiota, characteristic of older age, have been reported. These mechanisms are probably mediated by the age-related proinflammatory state [175,177,178].

Furthermore, it has been demonstrated in murine models that intestinal dysbiosis can alter neuromuscular transmission with a consequent promotion of muscle protein catabolism [179].

Thus, the administration of prebiotics, probiotics, and symbiotics, substances that improve microbiota health, has been proposed as a possible treatment for sarcopenia. Prebiotics are specific fermented ingredients that can induce changes in the composition and/or in the activity of the gastrointestinal microflora with a final beneficial effect for the host organism [79,180].

The actually available prebiotics are non-digestible oligosaccharides (i.e., inulin, oligofructose, (trans)galactooligosaccharides). They modulate the metabolism of the intestinal flora and show immunomodulatory properties [180]. Indeed, a study of Buigues [181] demonstrated that 13-week supplementation with prebiotics improved exhaustion and handgrip strength in older people.

Probiotics are instead viable microorganisms that can exert beneficial effects when administered in adequate quantities for reaching the intestine in an active state [182]. Probiotics modulate the intestinal microflora of the host by reducing microbial aberrancies and having an inflammatory effect [180].

The most used probiotics are Bifidobacteri and Lactobacilli [183]. Indeed, in animal studies, the administration of Lactobacillus reuteri, which modulates the transcriptional factor Forkhead Box N1 (FoxN1), was able to prevent cachexia in murine models of cancer [184]. Other probiotics, like *Faecalibacterium prausnitzii*, has anti-inflammatory proprieties [185] and in animal models, can improve the marker of oxidative stress [186].

Finally, symbiotics are a combination of prebiotics and probiotics exerting synergic effects [187].

Proteins, which are a known nutritional treatment for sarcopenia, represent a substrate for gut microbiota too.

Proteins increase microbiota diversity [188] and the number of protein-fermenting bacteria, which increase the bioavailability of dietary amino acids [175,188].

Short-chain fatty acids and secondary biliary salty acids, produced by microbiota, may counteract age-related muscle decline, too, thanks to their positive effects on muscle mitochondria. Moreover, they reduce host inflammation by decreasing TNFα-mediated immune responses and inflammasomes (i.e., NLRP3) [78].

We have to underline that the evidence of the effectiveness of prebiotics, probiotics, and symbiotics comes mainly from animal studies. Therefore, further research targeting specifically sarcopenic individuals is needed to recommend their routinely use.

## 3. Synergies between Nutritional and Physical Exercise Interventions

Inactivity is one of the main causes of sarcopenia [189] because it determines a resistance to muscle anabolic stimuli [190]. Therefore, the combination of nutritional interventions and physical exercise acts synergically to improve muscle health. Indeed, up to now it has been the most effective strategy for the management of sarcopenia.

The World Health Organization recommends performing at least 150 min/week of moderate-intensity physical activity or at least 75 min/week of vigorous-intensity physical activity or an equivalent combination of the two. An additional benefit can be obtained by increasing the amount of moderate-intensity physical activity to 300 min/week and by performing strengthening activities involving the major muscle groups on two or more days a week. Furthermore, for people with poor mobility, it is suggested to do exercises to enhance balance and prevent falls on three or more days per week [191]. Since sarcopenia involves muscles in the whole-body [7,192,193] it has been recently recommended to perform an holistic training involving all muscle groups [192].

It is interesting to note that when exercise is proposed to older people, they usually show a positive attitude and also enjoy the social component of the activities [194], even in the hospital setting [195].

The maximum effect of exercise is achieved within the first 3 h after training, but it can persist up to 24 h after the bout is over [196].

In particular, resistance training reduces insulin resistance, sensitizes muscles to other anabolic stimuli, and promotes mitochondrial biogenesis [78]. Therefore, resistance training is established to increase the synthesis of myofibrillar proteins in older people [197] with a consequent positive effect on muscle mass, strength, and performance [198,199,200,201,202,203,204]. These results are even appreciable at an extremely advanced age (i.e., centenarians) [205,206,207].

There are also potential indirect effects of exercise on gut microbiota that are noteworthy. By affecting intestinal mobility [208], exercise seems to reduce the dysbiosis, which negatively impacts muscle protein anabolism [209,210].

Here, we revise the major nutritional interventions that showed to have a synergic effect with physical exercise.

### 3.1. Protein/Amino Acid and Exercise

Exercise sensitizes the muscle to the anabolic actions of amino acids [211]. Indeed, the combination of protein/amino acid administration with physical exercise has proved to augment muscle protein anabolism compared to each intervention alone [212]. The synergistic effect is appreciable both in young and older people [213]. The sensitizing effect of exercise to amino-acid anabolic effects is maximal 3 h after the physical effort [211]. Therefore, proteins should be assumed 2–3 h after training [41]. Both resistance [73,197,214] and aerobic exercise [215,216] improve the protein anabolic stimulus. The effect is appreciable even if the intensity of exercise is only moderate [215]. Indeed, a metanalysis of 22 randomized controlled trials have confirmed that the combination of protein supplementation with resistance training has produced a greater increase in fat-free mass, type I and II muscle fiber cross-sectional area, and muscle strength compared to exercise alone [217]. These results have partially been endorsed by a more recent systematic review [218]. This review has included heterogeneous studies in terms of populations, duration, and dose of daily proteins. There have been negative findings in some of the included studies. These were mainly in older participants already with a sufficient protein and caloric intake and in people receiving soy proteins.

In summary, combination of protein/EAAs supplementation should be recommended in people who are deficient in association with physical exercise to prevent and reverse sarcopenia.

### 3.2. HMB and Exercise

The rationale for associating HMB supplementation and exercise is that HMB seems to promote the regenerative capacity of skeletal muscles after high-intensity exercise. Attenuation of markers of skeletal muscle damage after exercise were seen in case of administration of HMB [219]. Moreover, in one study, supplementation with HMB in association with strength training in older people increased more muscle mass and strength compared to exercise alone [220].

HMB effects are mainly appreciable in untrained people undergoing strenuous exercise, but also in trained people performing high physical stress training [95].

Indeed, a recent systematic review [221] has shown that the association of HMB plus resistance training enhances training-induced muscle mass and strength, attenuates markers of muscle damage, and improves markers of aerobic fitness. However, in another systematic review, the additional effect of HMB plus exercise was found only in one out of three randomized controlled trials for muscle mass and no additional effect was demonstrated for muscle strength and performance [218]. These negative findings can be explained by the fact that the suppression of proteolysis mediated by HMB may blunt the adaptation to training [95]. Moreover, a long period of pre-exercise supplementation may be necessary to achieve results [222].

### 3.3. Creatinine and Exercise

Considering the role of creatinine as energy buffer, it appears to be particularly useful in high-intensity exercise. The creatine-phosphocreatine system is highly used during these performances, so it can provide energy at a rapid rate.

The combination of Cr supplementation and resistance training increases IGF-1 at muscular level [127]. In turn, IGF-1 favors protein synthesis by activating the central mediator, PKB/AKT; and, subsequently, mTOR [223]. The final effect is an increase in muscle mass and strength, which can continue until 12 weeks after Cr withdrawal [224]. These results were confirmed both in young [225,226,227,228] and older [229,230,231] adults, though with some conflicting results [229,232,233,234]. Therefore, the PROT-AGE study group suggests supplementation only in older people who are deficient or at high risk of deficiency [41].

### 3.4. Vitamin D and Exercise

Vitamin D has a pivotal role on muscle tropism and function [235,236]. The effects of its deficiency on muscles are severe (extreme weakness and muscle pain) [236]. Anyway, in a recent review, no additional effect of vitamin D supplementation plus exercise was found for muscle mass and only conflicting results in terms of muscle performance [203]. Moreover, in the study of Bunout et al. [237], people were supplemented with a dose that was below (400 UI/daily) the recommended daily dose (800 UI/daily), while the study of Binder et al. [238] was considered of poor quality. It is reasonable that only individuals who are deficient would display an additional benefit of vitamin D supplementation over exercise. The deficit, causing muscle weakness and pain, would prevent the benefit from exercise training. Supplementation increases vitamin D receptor expression at muscle level with a positive effect in terms of muscle tropism and performance. Therefore, supplementation creates a positive background for the action of exercise in people who are deficient.

### 3.5. PUFA and Exercise

Results on the synergic effect of PUFA supplementation and exercise on muscle mass and performance are conflicting. In one study, the supplementation with PUFAs (fish oil) plus strength training produced great improvements in muscle strength and performance compared to exercise alone [239]. Anyway, another study has found that 12-week supplementation with α linoleic acid combined with resistance training had only marginal effects on muscle mass and strength [240]. A recent narrative review [241] has concluded that the synergic effect of the two interventions on muscle mass are still equivocal and conflicting about muscle function in older people. Therefore, there is insufficient evidence to recommend this intervention in sarcopenic individuals.

### 3.6. Practical Application

It is well known that older adults frequently have health-related problems, which may compromise the capacity to perform exercise tasks. Furthermore, since it has been shown that individual responses to nutrition/exercise interventions may be quite variable, a personalized approach to counteract muscle decline seem to be promising [242]. As mentioned above, resistance training is the most effective type of exercise to counteract and/or reverse sarcopenia. However, various training-related parameters (i.e., frequency, duration, intensity, volume, etc.), specifics for the older person, need to be considered in the implementation of exercise training programs [243].

Resistance training should be supervised both for compliance and safety (especially for those who are frail or sarcopenic) [244,245]. Furthermore, the time of intervention should be of at least three months to obtain significant improvement in muscle parameters (i.e., muscle strength and physical performance) [244] and exercise frequency should be of two or more nonconsecutive sessions per week [41]. An exercise duration of 10 to 15 min per session with eight repetition for each muscle group is considered sufficient to counteract muscle decline in healthy older people [41]. However, in frail and sarcopenic subjects, more time and repetitions may be needed to improve muscle parameters. A high-intensity resistance training (i.e., 80%–95% 1 repetition maximum) is recommended to induce maximum muscle hypertrophy or muscle fiber adaption [246,247]. Some authors reported that high-intensity resistance training is tolerated in older adults [206,248,249,250]. Unfortunately, this exercise intensity may not be achieved by frail subjects [251]. Nevertheless, lower intensities of exercise training (i.e., from 50% to 75%) may be sufficient to induce strength gains [192,243].

In a recent systematic review, Liao et al. [251] reported that protein supplementation, in addition to muscle strengthening exercise, is effective in promoting gain both in muscle mass and strength and enhancing physical performance in older adults with a high risk of sarcopenia or frailty.

In addition to increasing dietary protein intake to at least 1.2 g protein/kg of BW or providing supplements, it is recommended to supplement proteins or EEAs close after exercise sessions (i.e., 20 g of proteins) [41]. Nutritional status should be assessed before each intervention and the amount of proteins should be individually adjusted with regard to nutritional status, physical activity level, disease status, and tolerance [252].

## 4. Conclusions

The severe adverse consequences of sarcopenia and their impact on individuals and health systems make the treatment of this condition compelling. Importantly, the complexity of its pathogenesis represents a challenge for its management. Unfortunately, a contemporary pharmacological therapy is not yet available. However, nutritional interventions have shown to produce important beneficial effects on muscle parameters in older adults. Moreover, new dietary components with promising effects are emerging (i.e., gut microbiota manipulation). What is more, promotion of physical exercise according to the WHO guidelines is another efficacious modality considering the beneficial synergisms of nutritional and physical interventions.

In conclusion, sarcopenic individuals should assume 1–1.2 g proteins/kg body weight/day, with high content of EEAs (10–15 g) and leucine (at least 3 g) preferentially 2–3 h after exercise to maximize their anabolic effect. Older individuals who are deficient or at high risk of deficiency of vitamin D, creatinine, and HMB should be integrated.

Personalization of the diet and exercise programs according to patients’ needs remain the pivotal step for the treatment of sarcopenia. Moreover, preventive strategies to maximize the peak of muscle mass during the adulthood and reduce midlife muscle mass decline should be promoted configuring a life course approach to this condition, so that muscle function is preserved for as long as possible; and, thus positively affecting quality of life and health span.
